# Quantifying Dynamic Flow of Emergency Department (ED) Patient Managements: A Multistate Model Approach

**DOI:** 10.1155/2020/2059379

**Published:** 2020-12-03

**Authors:** Chung-Hsien Chaou, Te-Fa Chiu, Shin-Liang Pan, Amy Ming-Fang Yen, Shu-Hui Chang, Petrus Tang, Chao-Chih Lai, Ruei-Fang Wang, Hsiu-Hsi Chen

**Affiliations:** ^1^Department of Emergency Medicine, Chang Gung Memorial Hospital, Linkou and Chang Gung University College of Medicine, Taoyuan, Taiwan; ^2^Chang Gung Medical Education Research Centre, Chang Gung Memorial Hospital, Taoyuan, Taiwan; ^3^Institute of Epidemiology and Preventive Medicine, College of Public Health, National Taiwan University, Taipei, Taiwan; ^4^Department of Emergency Medicine, China Medical University Hospital and School of Medicine, China Medical University, Taichung, Taiwan; ^5^Department of Physical Medicine and Rehabilitation, National Taiwan University Hospital, National Taiwan University College of Medicine, Taipei, Taiwan; ^6^School of Oral Hygiene, College of Oral Medicine, Taipei Medical University, Taipei, Taiwan; ^7^Department of Parasitology, College of Medicine, Chang Gung University, Taoyuan, Taiwan; ^8^Emergency Department, Taipei City Hospital, Ren-Ai Branch, Taipei, Taiwan; ^9^Department of Emergency Medicine, Shin Kong Wu Ho-Su Memorial Hospital, Taipei, Taiwan; ^10^School of Medicine, Fu-Jen Catholic University, New Taipei City, Taiwan

## Abstract

**Background:**

Emergency department (ED) crowding and prolonged lengths of stay continue to be important medical issues. It is difficult to apply traditional methods to analyze multiple streams of the ED patient management process simultaneously. The aim of this study was to develop a statistical model to delineate the dynamic patient flow within the ED and to analyze the effects of relevant factors on different patient movement rates.

**Methods:**

This study used a retrospective cohort available with electronic medical data. Important time points and relevant covariates of all patients between January and December 2013 were collected. A new five-state Markov model was constructed by an expert panel, including three intermediate states: triage, physician management, and observation room and two final states: admission and discharge. A day was further divided into four six-hour periods to evaluate dynamics of patient movement over time.

**Results:**

A total of 149,468 patient records were analyzed with a median total length of stay being 2.12 (interquartile range = 6.51) hours. The patient movement rates between states were estimated, and the effects of the age group and triage level on these movements were also measured. Patients with lower acuity go home more quickly (relative rate (RR): 1.891, 95% CI: 1.881–1.900) but have to wait longer for physicians (RR: 0.962, 95% CI: 0.956–0.967) and admission beds (RR: 0.673, 95% CI: 0.666–0.679). While older patients were seen more quickly by physicians (RR: 1.134, 95% CI: 1.131–1.139), they spent more time waiting for the final state (for admission RR: 0.830, 95% CI: 0.821–0.839; for discharge RR: 0.773, 95% CI: 0.769–0.776). Comparing the differences in patient movement rates over a 24-hour day revealed that patients wait longer before seen by physicians during the evening and that they usually move from the ED to admission afternoon. Predictive dynamic illustrations show that six hours after the patients' entry, the probability of still in the ED system ranges from 28% in the evening to 38% in the morning.

**Conclusions:**

The five-state model well described the dynamic ED patient flow and analyzed the effects of relevant influential factors at different states. The model can be used in similar medical settings or incorporate different important covariates to develop individually tailored approaches for the improvement of efficiency within the health professions.

## 1. Introduction

Modern emergency medicine has undergone rapid growth over the past half a century. [1] Improvements in medical knowledge and diagnostic protocols have led to more competent emergency department (ED) systems that can manage a wide range of medical emergencies. As a result, the number of ED visits has continued to increase by an estimated 20% in the last ten years and 50% in the past two decades [2]. In addition to the upsurge in patient input, the median ED length of stay (LOS) has also been increasing consistently according to the literature [3]. For these reasons, ED crowding has become one of the most critical healthcare issues in many countries around the world, and the analysis of ED crowding, management processes, and efficiency is gaining increasing attention in the relevant research fields [4–6].

A large proportion of research regarding ED crowding has focused on LOS and patient arrival patterns [7–10]. Relevant influential factors associated with a more crowded ED or prolonged LOS can also be found in the literature [11–13]. Evidence also showed that common influential factors have different effects on different disposition groups of ED patients [14]. Due to advances in statistical modeling techniques applied in healthcare science, we are able to inspect more closely the current situation and the etiologies that cause ED crowding. In a previous review article, Wiler et al. introduced several modeling approaches that have been used to describe or predict ED management [6]. Some of these methods, such as regression-based methods, are useful for defining ED crowding, due to their ease of use; methods such as time series-based analysis are effective in predicting patient arrival patterns; and methods such as event-time analysis are able to analyze the influential factors affecting ED LOS. However, most approaches take a rather holistic view in analyzing ED crowding, and the detailed patient flow inside ED is still a black box. Very few methods enable the joint analysis of the whole management process within the ED system, and the knowledge of factors affecting different stages of the process is still limited.

Multistate Markov models belong to a special type of continuous time, discrete state, stochastic process, wherein the next step of clinical management depends only on the present state, not on sequence of events that preceded it, namely, the Markov property [15]. These types of models have been used in the healthcare research literature, especially when addressing disease progression, management processes, and transitions between different healthcare facilities [16–18]. Multistate modeling is very flexible in its design. Due to its mathematical formula-based nature, it has the strength to assess the effects of influential factors on different state transitions, such as the triage level, age, and disease entity [19]. In this article, the authors aimed to delineate dynamic ED patient management flow and to analyze the effects of relevant factors using a multistate Markov model.

## 2. Methods

### 2.1. Study Setting and Population

A retrospective administrative electronic data analysis was conducted. The study protocol was approved by the Chang Gung Medical Foundation Institutional Review Board (IRB No. 201601441B0) and was exempt from the requirement of obtaining informed consent. This study was conducted in the ED of the Linkou Chang Gung Memorial Hospital (LCGMH), a tertiary medical center with a 3,600 bed capacity in northern Taiwan. The annual number of ED visits was approximately 160,000 patients. The ED contains 120 treatment beds and 150 observation beds. The usual numbers of ED physicians on duty are approximately ten faculties and eight residents in the daytime and four faculties and five residents at night. The patient population consisted of local residents with general emergency conditions, as well as transfer cases from regional hospitals. The inclusion criterion was all patients who visited the LCGMH ED from January to December 2013. Patients with missing triage time, physician time, observation time, or leaving time were excluded from the analysis. Patients who died in the ED and patients with a missing endpoint were also excluded from the analysis. The patients were generally divided into three categories—trauma, adult nontrauma, and pediatric nontrauma—and managed in different areas within the ED. The demarcation between adult and pediatric nontrauma patients was 18 years of age.

### 2.2. Data Collection

Data were extracted from the hospital administrative electronic database. Discharged patients included those who were discharged by the primary ED physician, those who left without being seen, and those who left unnoticed. Admission patients included those admitted to the intensive care unit, those admitted to a ward, and those who were transferred to another hospital for admission. The outcomes of measurement were the states that each patient been to and the duration they stayed in each state. The time point variables included triage time recorded by the triage nurse; physician time recorded as the ED physician input the first order; observation/waiting for admission time, when the patient was moved to the observation area; and departure time recorded by the registration counter. Total LOS was defined as the time from triage to departure. Patient characteristic variables included age and gender. Disease and acuity variables included the patient category, triage level, whether the patient was transferred from another hospital, whether the patient was in an out-of-hospital cardiac arrest condition, and whether the patient was declared to be in a critical condition by the primary ED physician. Triage classification was sorted according to the Taiwan Triage Acuity System (TTAS), which is a five-level system [20]. The average admission rates for acuity levels from TTAS level I to level V are 62%, 77%, 20%, 17%, and 9%, respectively. A 24-hour fast track system is provided for low acuity adult nontrauma patients (TTAS level III–level V). All triage nurses were senior nurses who had attended a TTAS training program.

### 2.3. Model Building

At the study ED, most patients move from the triage nurse to physician management after a variable waiting time, with only a few exceptions who leave without being seen (LWBS) by a physician (<0.1%). After ED physician management, some patients leave the ED, either as instructed by the physician or against medical advice, and some patients are suggested to stay in the hospital for observation or admission. A proportion of patients who require admission are fortunate enough to have a ward bed available right away, and thus go directly to the ward. However, a larger proportion of patients who require admission have to stay in the observation room waiting for a ward bed. Therefore, some patients in the observation room only stay for observation and may be discharged before long; some patients are initially arranged for admission but are treated in the observation room for a short period of time and are then discharged due to improved condition; and others eventually have a bed available and leave the ED for the ward.

**Figure 1:**
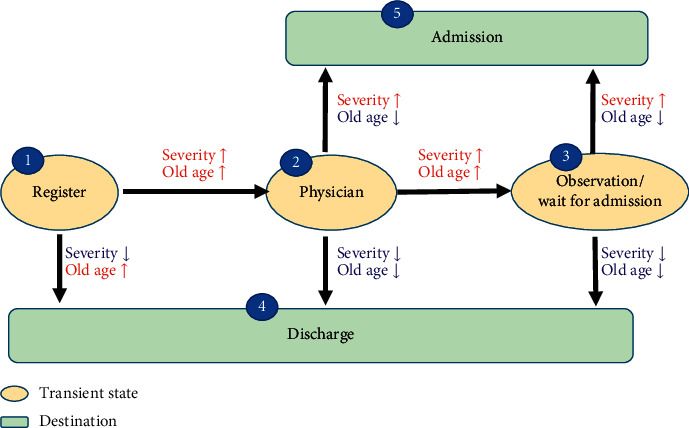
Five-state Markov model for the emergency department management process. The effects of covariates are also presented. An upward arrow indicates an accelerating effect on the patient movement rate, and a downward arrow indicates a decelerating effect.

Three expert panel meetings were held between March and August 2016 to build the model for this study. The members of the panel included three emergency physicians (CHC, CCL, and RFW) and two biostatisticians (THHC and AMFY). The aim was to build a parsimonious model that is both realistic in the clinical setting and easy to use. The states were divided into either intermediate or final, with the latter indicating states that once entered cannot be left. Four intermediate states—registration, triage, physician management, and observation—and three final destinations—admission, discharge, and death—were initially proposed. However, to simplify the model, death was discarded during the discussion process because only about 0.6% of total cases died in the ED. In addition, registration and triage were combined because the preliminary analysis of the data showed that the times of these two states were very close. Thus, the final model contained five states—triage, physician management, observation/waiting for admission, discharge, and admission—with the last two being final states. A diagram of the proposed five-state model is shown in Figure 1.

### 2.4. Multistate Markov Model and Adjustment of Influential Factors

The multistate Markov model is a type of continuous time, discrete state stochastic process model satisfying the Markov property, which is that predictions for a future move can be made based solely on its present state [15]. For instance, a patient who stayed in the ED for 10 hr and developed shifting pain and another patient who presented with initial Mcburney point rebound tenderness may have the same management flow after appendicitis was diagnosed, regardless of their length of stay or check-ups taken before the diagnosis was confirmed. The patient movement was presumed following an exponential distribution and governed by a rate parameter (*q*). The parameter represented the times of movements that occur per person per unit time. It can be shown that the mean time gap (sojourn time) before the next movement from state *i* to state *j* occurs is the reciprocal of the rate parameter, *q*_*ij*_.

In order to account for the daily variations in patient arrival and processing rates, a shift-based Markov model was further applied. One day was divided into six-hour intervals (0000–0600, 0600–1200, 1200–1800, and 1800–2400 hours), and the same model was used to estimate different patient movement rates in each time period. Individual covariates were incorporated into the model by setting the patient movement rate as functions of the covariates [21]. Exponentiating the regression coefficient results in a relative rate (RR) for the effect analyzed. The product of the movement rate and the RR is the new movement rate under the effect of the influential factor. A detailed model specification and likelihood function expression is given in supplementary material 1.

### 2.5. Statistical Analysis and Parameter Estimation

Mean and standard deviation (SD) were used to describe the central tendency and spread of continuous variables. Median and interquartile range (IQR) were used for continuous variables that obviously deviated from normal distribution. Comparisons of the variables between groups were made using an independent *t* test, analysis of variance, the Wilcoxon rank-sum test, or the chi-square test, as appropriate. The parameters of the Markov model were estimated using the Markov Chain Monte Carlo (MCMC) method. The MCMC method is a Bayesian iterative approach, which has the ability to combine previous experiences into prior settings. This is especially important in modeling regularly collected quality control parameters [22]. All analyses were performed using SAS statistical software version 9.3 (SAS Institute Inc., Cary, NC) [23]. A reported *p* value <0.05 was considered statistically significant.

## 3. Results

### 3.1. Descriptive Results

**Figure 2:**
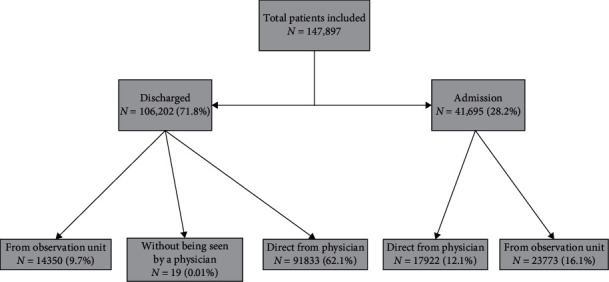
Diagram of patient flow with proportion.

Table 1 displays the descriptive results of a total of 149,468 enrolled patients included for analysis. Approximately 72% was eventually discharged from the ED, and the remaining 28% was admitted to the hospital. A detailed diagram of patient flow is sketched in Figure 2. The mean age of the study population was 39.8 (SD = 27.1) years, and 54.3% was male. When stratified into five 20-year age groups, the largest age group was patients under 20 years of age (29.2%). The largest proportion was noted in patients triaged as level III (59.6%), followed by triage level IV (17.7%), triage level II (15.2%), triage level I (5.83%), and triage level V (1.69%). The proportions of the different patient categories—trauma, adult nontrauma, and pediatric nontrauma—were 17.5%, 59.4%, and 23.1%, respectively. In terms of time variables, median total LOS was 2.12 (IQR = 6.51) hours, and median triage to physician and physician to the observation room times were 0.16 (IQR = 0.15) and 1.50 (IQR = 1.17) hours, respectively. The rest of the median patient movement times between states are shown in Table 1.

### 3.2. Estimation of Patient Movement Rate Parameters and Covariate Effects

The rates of patient movement are shown in Table 2. Recall that the higher the movement rate, the shorter the time spent in waiting for a movement between two states. Put it in another way. The lower the movement rate, the lower the chance of having the corresponding movement. The results shown in Table 2 show the rate or the odds of each movement in Figure 2. From the entry of registry, it was rather faster for triage to physician (MR = 4.224 (95% CI: 4.204–4.247)), but it is very unlikely to see the movement from triage to departure (MR=0.0005 (95% CI: 0.0003–0.0008)). The latter is often referred to as the LWBS patients, and the small estimate confirms the low LWBS rate in the study hospital. The results also showed that, after triage, in a steady ER system with 60 patients in the treatment bed area and 100 patients in the observation area, how many patients have each kind of transition between two states in an hour. About 6 patients would have the movement from the physician to the observation room in an hour (MR = 0.099, 95% CI: 0.098–0.100). Around 14 patients (MR = 0.235, 95% CI: 0.233–0.236) move from physician to discharge and only 1 patient (0.0114, 95% CI: 0.0112–0.0116) had discharged from the observation area. Around 3 (MR = 0.046, 95% CI: 0.045–0.047) patients had the movement of physician to admission and 2 patients or so (MR = 0.0189, 95% CI: 0.0186–0.0191) had the movement of observation area to admission.

The effects of the covariates are also displayed in Table 2. Triage is an important variable for ED patients because the highest acuity patients should be seen as quickly as possible. In Table 2, it is clear that the RR of the triage level on the patient movement rates of triage to the physician (RR on *q*_12_ = 0.962, 95% CI: 0.956–0.967), physician to the observation room (RR on *q*_23_ = 0.673, 95% CI: 0.666–0.679), physician to admission (RR on *q*_25_ = 0.757, 95% CI: 0.745–0.766), and observation room to admission (RR on *q*_35_ = 0.842, 95% CI:0.826–0.856) were all less than 1, indicating a slowering effect. In other words, patients with lower acuity levels had to wait longer for physicians and admission beds. On the other hand, the RRs of the triage level on the patient movement rates of physician to discharge (RR on *q*_24_ = 1.891, 95% CI: 1.881–1.900) and observation to discharge (RR on *q*_34_ = 1.650, 95% CI: 1.619–1.677) were all greater than 1, indicating that the lower acuity patients went home sooner.

The estimated number of patients moved from one stage to another stage in a steady ER is also provided in Table 2. Because the movement rate is estimated per person-hour, the more the people stay in a stage, the more people are likely to move to the next stage. Regarding the effect of the age group on the patient movement of each state, it can be seen that the age group has RRs greater than 1 on patient movement rates of triage to the physician (RR on *q*_12_ = 1.134, 95% CI: 1.131–1.139) and the physician to the observation room (RR on *q*_23_ = 1.549, 95% CI: 1.539–1.562). These results suggest that older patients were seen by physicians or recommended for admission more quickly. On the other hand, the RRs for patient movement rates of the physician to discharge (RR on *q*_24_ = 0.773, 95% CI: 0.769–0.776), the physician to admission (RR on *q*_25_ = 0.830, 95% CI: 0.821–0.839), the observation room to discharge (RR on *q*_34_ = 0.619, 95% CI: 0.609–0.628), and the observation room to admission (RR on *q*_35_ = 0.910, 95% CI: 0.899–0.921) were all less than 1, implying that older patients spent more time waiting for the final state.

### 3.3. Results of the Shift-Based Five-State Markov Model

To account for changes in patient movement rates over time during a day, a shift-based Markov model was further applied. The results of estimated patient movement rates for each six-hour interval—night (0000–0600 h), morning (0600–1200 h), afternoon (1200–1800 h), and evening (1800–2400 h)—are presented in supplementary material 2, where some of the differences in patient management flows during different time periods within a 24-hour day could be seen. For instance, the patient movement rate from triage to the physician was significantly slower in the evening (*q*_12_=3.88723.8872, 95% CI: 3.8543–3.9232), meaning that staffing during this time period is relatively short. Another example is that the patient movement rates from the observation room to the ward were much faster in the afternoon (*q*_35_=0.0390, 95% CI: 0.0383–0.0398) and evening (*q*_35_=0.0327, 95% CI: 0.0321–0.0333) compared with night (*q*_35_=0.0012, 95% CI: 0.0011–0.0013) and morning (*q*_35_=0.0069, 95% CI: 0.0066–0.0072). This is because ward patients have to be discharged before ED patients can be admitted, and beds are usually available afternoon.

## 4. Discussion

Although multistate models are used widely in healthcare science, it has rarely been used to analyze the ED patient management process. The characteristics of the multistate Markov model are that it is flexible to use; it has the ability to cope with individual differences and pattern descriptions and to analyze covariate effects at the same time, with the cost of a rather complicated computation [15]. In the current study, the model estimated the patient movement rates between the triage, physician management, observation room, admission, and discharge states. The model also demonstrated that in the study ED, higher acuity accelerated the pace to physician management and admission, but decelerated the speed to discharge. In addition, older patients waited less time before being seen by a physician, but more time before being admitted or sent home, which might be because a greater number of exams or image studies needed to be performed in the ED. When comparing differences in patient movement rates among different time points within a 24-hour day, wait time before physician management was longest in the evening, and patient flow from the ED to admission was fastest afternoon.

Various other statistical approaches have been proposed in previous studies to address the issue of ED crowding, and each different approach has its own strengths and weaknesses. For instance, regression-based methods are frequently used, convenient tools for defining or seeking factors associated with crowding [24, 25]. These models are relatively easy to use, but they are very dependent on the covariates selected. Time series-based approaches are another type of modeling method that focuses on forecasting patient arrival number based on recent historical data. Due to the nature of this method, most of its use is targeted to the input rather than the throughput of patients [26, 27].

One similar approach to describe the ED process statistically is through queueing models or networks. Queueing models describe incoming patient flow versus physician staffing policy by making assumptions regarding arrival rate and service times. Good examples of the use of queueing models in the ED are available in the literature [28–30]. However, several difficulties have been encountered when applying queueing models due to their underlying assumptions, such as assumption of first-come first-served, assumption of no patient leave without treatment complete, and assumption of no other unscheduled delays other than the queues [31–34]. Take first-come first-serve assumption, for example, an ED physician is managing a severe sepsis patient and a minor trauma patient and happens to have time for another minor fever patient with flu-like symptoms. The doctor does some history taking and a physical examination and then discharges the influenza patient home with medication. This situation may be complicated when dealing with a queueing model, but it is simple when using a multistate model, as once the patients enter the same state, their departure from the state is modeled by probability, regardless of their previous status or arrival sequence.

Another more advanced approach is the discrete event simulation (DES). Instead of specifying underlying mathematical formula and likelihood, DES models a network of interdependent discrete events through computer simulation [35, 36]. The model is robust and flexible, tolerates detailed constraint settings, and had been widely used in the process planning or optimization within the ED in recent decades [37–39]. Previously, this approach was regarded as time-consuming and expertise required, but had been gradually overcome by the improvement of computational capabilities and commercially available softwares [40]. Compared with multistate Markov models, DES has been shown to have better prediction accuracies [41], but may not be as convenient when incorporating influential factors and estimating its effect on management flows.

Multistate models dissect the ED patient management process into different segments, analyze the patient movement rates of each state simultaneously, and quantify relevant covariate effects. Tailored improvement methods can then be derived according to individual ED settings. In EDs with slow triage to physician patient movement rates and policies such as physician at triage [42, 43] or in-room registration [44] might help to shorten the ED LOS. In EDs where patient movement from the observation room to admission is notably slow, efforts could be put into improving the hospital level daily admission–discharge ratio [45] or setting up an emergency medicine ward [46] or acute medical admission units [47]. Finally, if an ED has a large proportion of low acuity patients but the physician to discharge movement rate is decreasing, creating a rapid medical assessment program [48] or fast tracks for nonadmission patients [49] might be effective.

**Figure 3:**
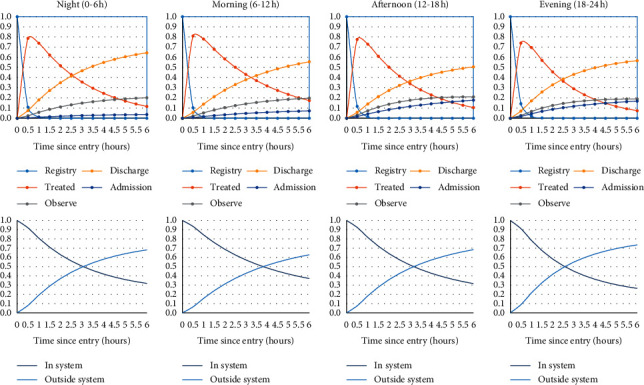
Predictive dynamic distribution of a patient who arrives at 0000, 0600, 1200, and 1800, using the estimated parameters of six-hour time periods. Upper, possibility of being in different states within the next six hours. Lower, probability of being inside or outside the ED system.

Another possible use of the analyzed results is to make predictive dynamic distributions of the patients through the estimated parameters. For one patient who arrives at 0000, 0600, 1200, or 1800, the probability of being in the different states is calculated using the estimated patient movement rates in supplementary material 2 and illustrated in Figure 3 (upper). As can be seen, the highest probability of being treated by the physician is approximately 0.5 hours after triage without significant variation between different time intervals. The probability of being discharged increases gradually till six hours later, with the highest of 65% chance at midnight. These probabilities could be used with aggregate patient data at the administrative level, as well as with individual data for prediction after implementing relevant covariates such as the age group, triage level, and patient category. To simplify the results further, the probability of being inside or outside of the ED system during these four time intervals can be determined as shown in Figure 3 (lower). The figure shows that six hours after entry, the probability of still in the ED system ranges from 28% in the evening to 38% in the morning. Surveillance system can then be built up based on credible intervals of estimates from regularly collected data. Once the patient movement rate decreased to a certain cutpoint, then adding additional manpower would be indicated.

## 5. Limitations

First, this research is a single-center study. When applying the results to another ED with quite a different patient population and hospital setting, the generalizability of the results might be limited. Nevertheless, the model was demonstrated to be useful in understanding patient management flows within the ED as well as the effects of relevant covariates on each of the state transitions. When used as a tool for process monitoring, tailored improvement plans can be developed according to the findings of individual EDs. Further study is aimed at implementing this model in different hospital settings and validating the model by using it as a prediction tool. Second, because of the number of parameters that were estimated jointly, we treated the age group and triage levels as continuous variables. In real practice, these factors should be treated as categorical to make a more precise estimation.

## 6. Conclusion

In this study, a five-state Markov model was used to model the dynamic management process among ED patients and assess the effects of relevant influential factors. When used in the study ED, the results showed that patients with lower acuity went home faster but had to wait longer for physicians or admission beds. Older patients were seen by physicians more rapidly, but spent more time waiting for the final states. In comparing patient movement rates among different time periods, it was found that patients waited longer before seen by physicians during the evening, and that they were moved from the ED to admission most often afternoon. The model can be used in similar medical settings or incorporate different important covariates to develop individually tailored approaches for the improvement of efficiency within the health professions.

## Figures and Tables

**Table 1:** Descriptive results of the patients included the study, presented as count (%) unless stated otherwise (*n* = 147,897).

Age^*∗*^	39.7	(27.1)

Age group		
<20	43,318	(29.3)
20–40	31,694	(21.4)
40–60	33,867	(22.9)
60–80	28,501	(19.3)
>80	10,527	(7.12)

Male sex	80,260	(54.3)
Patient entity		
Adult nontrauma	87,494	(59.2)
Pediatric nontrauma	34,336	(23.2)
Trauma	26,067	(17.6)

Triage level		
Level 1	8,253	(5.58)
Level 2	22,483	(15.2)
Level 3	88,546	(59.9)
Level 4	26,138	(17.7)
Level 5	2,477	(1.67)

Final disposition		
Discharged by physician	101,972	(69.0)
Left unnoticed	509	(0.34)
Against medical advice discharge	3,702	(2.50)
Left without being seen	19	(0.01)
Admission to intensive care unit (ICU)	4,680	(3.16)
Admission to ward	36,121	(24.4)
Transferred to another hospital	894	(0.60)

Time variables (hr)^$^		
Total length of stay (*n* = 147,897)	2.12	(6.51)
Triage to physician (*n* = 147,878)	0.16	(0.15)
Physician to observation room (*n* = 38,123)	1.50	(1.17)
Triage (directly) to departure (*n* = 19)	1.86	(2.82)
Physician (directly) to discharge (*n* = 91,833)	1.01	(1.38)
Observation room to discharge (*n* = 14,350)	11.1	(33.0)
Physician (directly) to admission (*n* = 17,922)	4.33	(6.04)
Observation room to admission (*n* = 23,773)	25.9	(46.9)

^*∗*^Presented as mean (standard deviation). ^$^Presented as median (interquartile range).

**Table 2:** Estimated rates of patient movement rates (per person-hour) and the effects of the age group and triage level from the five-state Markov model.

Patient movement	Movement rate		Number of movement in a steady ER^#^	Effect of the triage level		Effect of the age group	
	Estimate	95% CI		RR	95% CI	RR	95% CI
Triage ⟶ physician	4.224	(4.204–4.247)	—	0.962^*∗*^	0.956–0.967	1.134^*∗*^	1.131–1.139
Physician ⟶ observation room	0.099	(0.098–0.100)	6	0.673^*∗*^	0.666–0.679	1.549^*∗*^	1.539–1.562
Triage (directly) ⟶ departure	0.0005	(0.0003–0.0008)	0	1.481	0.974–1.962	1.174	0.840–1.618
Physician (directly) ⟶ discharge	0.235	(0.233–0.236)	14	1.891^*∗*^	1.881–1.900	0.773^*∗*^	0.769–0.776
Observation room ⟶ discharge	0.011	(0.011–0.012)	1	1.650^*∗*^	1.619–1.677	0.619^*∗*^	0.609–0.628
Physician (directly) ⟶ admission	0.046	(0.045–0.047)	3	0.757^*∗*^	0.745–0.766	0.830^*∗*^	0.821–0.839
Observation room ⟶ admission	0.019	(0.018–0.019)	2	0.842^*∗*^	0.826–0.856	0.910^*∗*^	0.899–0.921

^#^After triage and in a steady ER system with 60 patients in the treatment bed area and 100 patients in the observation, the number of patients for each kind of movement between two states in an hour, rounded to integer. ^*∗*^Statistically significant. RR, relative rate. The reciprocal of the patient movement rate is the mean time gap before the next patient movement occurs. An effect of greater than 1 represents an accelerating effect on the corresponding movement.

## Data Availability

The deidentified data used to support the findings of this study are available from the corresponding author upon request.

## Supplementary Materials

Supplementary Materials Supplementary material I: model specification and likelihood function of a five-state Markov model. Supplementary material II: results of the patient movement rates (per person-hr) estimation from the five-state Markov model.
