# Supplementary material for: Quantifying Dynamic Flow of Emergency Department (ED) Patient Managements: A Multistate Model Approach
Source: Emerg Med Int. 2020 Dec 3;2020:2059379. doi: 10.1155/2020/2059379 (PMC7737449; doi:10.1155/2020/2059379)
Supplement: Supplementary Materials — Supplementary material I: model specification and likelihood function of a five-state Markov model. Supplementary material II: results of the patient movement rates (per person-hr) estimation from the five-state Markov model. [file 2059379.f1.zip › 2059379.f1/Supplementary material I_model specification.docx]

# Supplementary material I.

**Model Specification and Likelihood Function of a Five-State Markov Model**

In the five-state Markov model proposed in the current study, the transition of states, such as from state *i* to state *j*, is modeled through transition rates, presumed to be an exponential distribution, and governed by a rate parameter, denoted by $q_{ij}$. It can be shown that the mean sojourn time for staying in state *i* before entering state *j* is the reciprocal of $q_{ij}$. Because the transitions of different patients can occur between different states simultaneously, transition rates are presented in matrix form. The transition matrix *Q* for the five-state Markov process described above could be expressed as

$$Q=\left[ \begin{matrix} \begin{matrix} -(q_{12}+q_{14}) \\ 0 \\ \begin{matrix} 0 \\ 0 \\ 0 \end{matrix} \end{matrix} & \begin{matrix} q_{12} \\ -(q_{23}+q_{24}+q_{25}) \\ \begin{matrix} 0 \\ 0 \\ 0 \end{matrix} \end{matrix} & \begin{matrix} \begin{matrix} 0 \\ q_{23} \\ \begin{matrix} -(q_{34}+q_{35}) \\ 0 \\ 0 \end{matrix} \end{matrix} & \begin{matrix} q_{14} \\ q_{24} \\ \begin{matrix} q_{34} \\ 0 \\ 0 \end{matrix} \end{matrix} & \begin{matrix} 0 \\ q_{25} \\ \begin{matrix} q_{35} \\ 0 \\ 0 \end{matrix} \end{matrix} \end{matrix} \end{matrix} \right]$$

The zeros in matrix Q indicate that no transition occurred between the two states.

To adjust for the effects of individual covariates on multi-state transition rates, individual covariates were incorporated into the model by setting the transition rate as functions of the above covariates:

$$q_{ij}^{m}=q_{ij0}exp(\beta_{ij}X^{m})$$

where $q_{ij0}$ is the baseline transition rate of $q_{ij}$, $X^{m}$ is the individual covariate for patient *m,* and $\beta_{ij}$ is the coefficient for effect X on $q_{ij}$. Exponentiating the beta coefficient results in a Relative Rate (RR) for effect X.

The corresponding transition probability matrix, which provides the probability of transition during a time *t*, could be derived using Q from a forward Kolmogorov equation, in terms of transition rate parameters. Let $P_{ii}(t)$ represents the probability that a patient will stay in the same state *i* until time *t*. For instance, $P_{11}(t)$ could be derived and expressed as:

$$P_{11}(t)= e^{-(q_{12}+q_{14})\cdot t}$$

Similarly, $P_{23}(t)$ could be derived and expressed as:

$$P_{23}= -\left( \frac{\lambda2*e^{\left( -\lambda2 - \mu2 - \mu4 \right)*t}}{\lambda_{2} +\mu_{2}-\mu_{3}+\mu_{4}-\mu_{5}} \right)+ \frac{\lambda2*e^{\left( -\mu3 - \mu5 \right)*t}}{\lambda_{2} +\mu_{2}-\mu_{3}+\mu_{4}-\mu_{5}}$$

The likelihood function for transition from state *i* to state *j* at time *t* can be expressed as $P_{ii}(t)\cdot q_{ij}$. Where *s* represents patients, *s*=1,2,…,n, and *c* represents transitions within the same patient, *c*=1,2,…,$m_{s}$, and $m_{s}$ represents the total transition of the *s*th patient, the full likelihood function could then be written as

|  | $L=\prod_{s=1}^{n} \prod_{c=1}^{m_{i}} P_{ii}\left( t \right)\cdot q_{ij}$ |  |
| --- | --- | --- |
