# Supplementary material for: Quantifying Dynamic Flow of Emergency Department (ED) Patient Managements: A Multistate Model Approach
Source: Emerg Med Int. 2020 Dec 3;2020:2059379. doi: 10.1155/2020/2059379 (PMC7737449; doi:10.1155/2020/2059379)
Supplement: Supplementary Materials — Supplementary material I: model specification and likelihood function of a five-state Markov model. Supplementary material II: results of the patient movement rates (per person-hr) estimation from the five-state Markov model. [file 2059379.f1.zip › 2059379.f1/Supplementary material II_estimated patient movement rates.docx]

# Supplementary material II. Results of the patient movement rates (per person-hr) estimation from the Five-State Markov model.

| **Patient movements** | **Night (0-6h)** | | |  | **Morning (6-12h)** | | |  | **Afternoon (12-18h)** | | |  | **Evening (18-24)** | | |
| --- | --- | --- | --- | --- | --- | --- | --- | --- | --- | --- | --- | --- | --- | --- | --- |
|  | **Rate** | **95% CI** | |  | **Rate** | **95% CI** | |  | **Rate** | **95% CI** | |  | **Rate** | **95% CI** | |
| **Triage → physician** | 4.4331 | 4.3737 | - 4.4875 |  | 4.5385 | 4.4929 | - 4.5869 |  | 4.2892 | 4.2486 | - 4.3288 |  | 3.8872 | 3.8543 | - 3.9232 |
| **Physician → observation room** | 0.0873 | 0.0855 | - 0.0894 |  | 0.0778 | 0.0758 | - 0.0796 |  | 0.1158 | 0.1138 | - 0.1178 |  | 0.1100 | 0.1079 | - 0.1118 |
| **Triage (directly) → departure** | 0.0001 | 0.0000 | - 0.0011 |  | 0.0002 | 0.0000 | - 0.0008 |  | 0.0004 | 0.0002 | - 0.0010 |  | 0.0009 | 0.0005 | - 0.0016 |
| **Physician (directly) → discharge** | 0.2705 | 0.2672 | - 0.2739 |  | 0.2007 | 0.1980 | - 0.2038 |  | 0.2128 | 0.2102 | - 0.2156 |  | 0.2739 | 0.2705 | - 0.2773 |
| **Observation room → discharge** | 0.0047 | 0.0045 | - 0.0049 |  | 0.0121 | 0.0117 | - 0.0125 |  | 0.0209 | 0.0204 | - 0.0214 |  | 0.0093 | 0.0090 | - 0.0097 |
| **Physician (directly) → admission** | 0.0153 | 0.0145 | - 0.0161 |  | 0.0248 | 0.0236 | - 0.0259 |  | 0.0624 | 0.0610 | - 0.0638 |  | 0.0697 | 0.0681 | - 0.0714 |
| **Observation room → admission** | 0.0012 | 0.0011 | - 0.0013 |  | 0.0069 | 0.0066 | - 0.0072 |  | 0.0390 | 0.0383 | - 0.0398 |  | 0.0327 | 0.0321 | - 0.0333 |
